# Diagnostic performances of D-dimer, prothrombin time, and red blood cell distribution width for coronary artery lesion in children with acute stage Kawasaki disease

**DOI:** 10.3389/fped.2023.1141158

**Published:** 2023-07-25

**Authors:** Qi-Gai Yin, Jing Zhou, Qin Zhou, Lu Shen, Mei-Yu Zhang, Yan-Hui Wu

**Affiliations:** ^1^ Department of Pediatrics, The People's Hospital of Suzhou New District, Suzhou, China; ^2^ Department of Pediatrics, Lianyungang Clinical Medical College, Nanjing Medical University, Lianyungang, China

**Keywords:** coronary artery lesion, D-dimer, Kawasaki disease, prothrombin time, red

## Abstract

**Aim:**

To evaluate the performances of D-dimer, prothrombin time (PT), and red blood cell distribution width (RDW) for the diagnosis of coronary artery lesion (CAL) in acute stage Kawasaki disease (KD).

**Methods:**

Between January 2018 and January 2021, a total of 102 children with acute stage KD were included in this retrospective study. Among them, 36 KD children with CAL were divided into the CAL group, and 66 KD children without CAL were divided into the NCAL group. Independent predictors of CAL in acute stage KD were identified by using univariate and multivariate logistic regression analysis. Spearman correlations were used to evaluate the association between CAL in acute stage KD and different indicators. The diagnostic performance of different indicators for CAL in acute stage KD was analyzed by the receiver operating characteristic (ROC) curve.

**Results:**

Compared with the NCAL group, children in the CAL group had significantly higher white blood cell count, lymphocyte count, platelet count, D-dimer, and RDW levels, but lower PT levels (all *p *< 0.05). Logistic regression analysis revealed that D-dimer (OR = 1.0, 95% CI: 1.004–1.012, *p *< 0.001), PT (OR = 0.4, 95% CI: 0.2–0.8, *p *= 0.01), and RDW (OR = 7.0, 95% CI: 2.6–19.2, *p *< 0.001) were independent predictors of CAL in children with acute stage KD. CAL showed a positive correlation with D-dimer (*r* = 0.4, *p *< 0.001) and RDW (*r* = 0.5, *p *< 0.001), and had a negative association with PT (*r* = −0.2, *p *< 0.05). The ROC curve analysis showed that the combination of the three indicators had the highest diagnostic performance for CAL in acute stage KD with an area under the curve (AUC) of 0.922 (sensitivity, 86.1%; specificity, 89.4%), compared with D-dimer (AUC = 0.736), PT (AUC = 0.640), and RDW (AUC = 0.819) alone.

**Conclusion:**

A combination of D-dimer, PT, and RDW may help predict CAL in children with acute stage KD.

## Introduction

Kawasaki disease (KD) is an acute systemic vasculitis syndrome that occurs in infants and children and is now the most common cause of acquired pediatric heart disease in developed countries (1). Ethnic variation in KD incidence rates was considerable, with the highest incidence rates among Asian or Pacific Islanders (29.8 per 100,000 children under 5 years of age) and the lowest incidence rates among Caucasian children (13.7 per 100,000) (2). Coronary artery lesion (CAL) is the most feared sequelae of KD, occurring in 20%–25% of untreated children (3). The presence of CALs in children with KD might lead to coronary aneurysms, and even myocardial infarction and sudden death (4). As the etiology of KD is unknown, there is no curative treatment to prevent CALs in children with KD (5, 6). Currently, the evaluation of CALs in KD relies on echocardiography, but the diagnostic accuracy is low due to the limited visibility of the distal coronary arteries (7, 8). Besides, some potential biomarkers have been reported to be associated with CAL in KD, but there are relatively few reliable predictors of CAL in acute stage KD (9–13), and further laboratory studies are necessary.

Evidence has showed that patients with higher serum D-dimer level, prolonged prothrombin time (PT), or increased red blood cell distribution width (RDW) had adverse outcomes of systemic inflammatory conditions (14–16). Recently, the role of inflammation in cardiovascular disease have been underlined, suggesting that lowering the burden of inflammation can reduce future cardiovascular events (17). Consistently, it has been shown that these indicators play important roles in the diagnosis or prognosis of cardiovascular disease. For example, a prospective cohort study showed that low PT levels were a risk factor related to major adverse cardiovascular events (18). In addition, the role of D-dimer in the diagnosis of clinical disorders of venous thromboembolism, pulmonary emboli, coronary artery disease, and disseminated intravascular coagulation has been established (19). Recently, several studies have shown that elevated RDW is correlated with worse outcomes in a variety of clinical conditions, including thromboembolic events, and cardiovascular disease (20–22). However, the diagnostic performances of D-dimer, PT, and RDW for CAL in KD have not been studied yet. Therefore, the retrospective study was conducted to evaluate the performances of D-dimer, PT, and RDW for the diagnosis of CAL in children with acute stage KD.

## Materials and methods

### Patients

Between January 2018 and January 2021, 102 children with acute stage KD treated at Lianyungang Hospital of Nanjing Medical University were included in this retrospective study. The study was approved by the ethics committee of the Lianyungang Hospital of Nanjing Medical University (number, LW-20210811001–01), and conducted following the Declaration of Helsinki. Informed consent was obtained from the patient's parents.

Children who were aged ≤14 years, and were newly diagnosed with acute stage KD (course of the disease, 1–11 days) according to the American Heart Association guideline (23), were eligible for the study. This study excluded children with incomplete clinical data, blood system disease or rheumatic immune system disease, congenital heart disease, and inherited metabolic disorders, as well as had received drug therapy (such as intravenous immunoglobulin and aspirin).

### Echocardiography measurements

Echocardiography was performed by experienced sonographers to detect CAL before intravenous immunoglobulin administration. The ultrasound instrument GE Vivid E95 (GE Vingmed Ultrasound, Horten, Norway) with a frequency of 1.5–4.5 MHz was used in this study. CAL was defined according to internal lumen diameter of >3.0 mm in children <5 years of age, >4 mm in children of ≥5 years of age; internal diameter of a segment measuring ≥1.5 times that of an adjacent segment; and coronary dimensions adjusted for body surface area (z scores) of ≥2.0 (24). According to the echocardiography results, children were divided into the CAL group and the non-CAL (NCAL) group.

### Data collection

Information on the children's demographic characteristics, echocardiography, and laboratory test results were collected retrospectively. Laboratory data including platelet count (PLT), lymphocyte count (L), neutrophil count (N), white blood cell count (WBC), C-reactive protein (CRP), prothrombin time (PT), activated partial thromboplastin time (APTT), mean platelet volume (MPV), platelet distribution width (PDW), erythrocyte sedimentation rate (ESR), D-dimer, red blood cell distribution (RDW), alanine aminotransferase (ALT), and aspartate aminotransferase (AST) were collected. Blood samples were collected before intravenous immunoglobulin administration.

### Statistical analyses

The Shapiro-Wilk test was used to check the distribution of variables. Quantitative data were expressed as means with standard deviations or median with interquartile range, and were compared using Student's *t*-test (normally distributed data) or Mann-Whitney *U*-test (skewed distributed data). Qualitative data were expressed as numbers and percentages, and were compared using *χ*2 tests. Univariate and multivariate logistic regression analysis was used to identify independent predictors of CAL in acute stage KD. Spearman correlations were used to investigate the associations between indicators and CAL in acute stage KD. To evaluate the performance of different indicators in discriminating CAL in acute stage KD, the area under the curve (AUC) using receiver operating characteristic (ROC) analysis was performed. Statistical significance was set at *p *< 0.05. The SPSS software (version 19.0, SPSS Institute. IL., USA) was used for statistical analysis.

## Results

### Comparison of baseline characteristics among the two groups

Between January 2018 and January 2021, a total of 102 children with acute stage KD (CAL group, *n* = 36; NCAL group, *n* = 66) were included in this retrospective study. The differences in baseline characteristics among the two groups are shown in Table 1. There were no significant differences in age, gender, fever duration, N, CRP, ESR, PDW, MPV, ALT, and AST among the two groups (all *p *> 0.05). The levels of WBC ([18.6 ± 5.3] × 10^9^/L vs. [14.7 ± 3.0] × 10^9^/L), L ([5.6 ± 3.1] × 10^9^/L vs. [3.6 ± 2.1] × 10^9^/L), PLT ([430.5 ± 133.8] × 10^9^/L vs. [369.4 ± 106.4] × 10^9^/L), D-dimer (0.5 ± 0.2 mg/L vs. 0.3 ± 0.2 mg/L), and RDW (13.4 ± 0.9% vs. 12.5 ± 0.6%) in the CAL group were significantly higher than that in the NCAL group (all *p* < 0.05). Besides, children in the CAL group had lower PT levels than those in the NCAL group (13.5 ± 1.5 s vs. 14.2 ± 1.3 s, *p *< 0.05).

**Table 1:** Baseline characteristics of the children with acute stage KD.

	CAL group (*n* = 36)	NCAL group (*n* = 66)	*P*-value
Age (years), median (IQR)	25.5 (12.3, 49.5)	24.0 (15.5, 35.3)	0.5
Male (*n*, %)	26 (72.2)	43 (65.2)	0.5
Fever duration (days), median (IQR)	6.0 (5.0, 7.8)	6.5 (5.0, 8.0)	0.7
WBC (×10^9^/L), mean ± SD	18.6 ± 5.3	14.7 ± 3.0	<0.001
N (×10^9^/L), mean ± SD	11.0 ± 4.1	10.3 ± 3.3	0.4
L (×10^9^/L), mean ± SD	5.6 ± 3.1	3.6 ± 2.1	<0.001
RDW (%), mean ± SD	13.4 ± 0.9	12.5 ± 0.6	<0.001
PLT (×10^9^/L), mean ± SD	430.5 ± 133.8	369.4 ± 106.4	0.01
CRP (mg/L), mean ± SD	83.3 ± 60.0	76.3 ± 51.3	0.5
ESR (mm/h), mean ± SD	43.1 ± 16.5	40.4 ± 20.4	0.5
PDW (%), median (IQR)	15.6 (15.3, 15.9)	15.6 (15.4, 15.8)	0.4
MPV (fL), median (IQR)	8.7 (7.8, 9.3)	8.4 (7.9, 9.3)	0.5
ALT (U/L), median (IQR)	31.0 (16.5, 68.0)	35.5 (14.8, 96.8)	0.7
AST (U/L), median (IQR)	39.5 (25.3, 54.3)	42.0 (28.0, 71.8)	0.3
PT (s), mean ± SD	13.5 ± 1.5	14.2 ± 1.3	0.02
APTT (s), mean ± SD	33.9 ± 5.5	34.8 ± 6.6	0.5
D-dimer (mg/L), mean ± SD	0.5 ± 0.2	0.3 ± 0.2	<0.001

KD, Kawasaki disease; CAL, coronary artery lesion; NCAL, non-CAL; IQR, interquartile range; SD, standard deviation; WBC, white blood cell count; N, neutrophil count; L, lymphocyte count; RDW, red blood cell distribution; PLT, platelet count; CRP, C-reactive protein; ESR, erythrocyte sedimentation rate; PDW, platelet distribution width; MPV, mean platelet volume; ALT, alanine aminotransferase; AST, aspartate aminotransferase; PT, prothrombin time; APTT, activated partial thromboplastin time.

### Logistic regression analysis

Risk factors associated with CAL in acute stage KD were further analyzed (Table 2). Univariate logistic regression analysis showed that WBC (OR = 1.3, 95% CI: 1.1–1.4, *p *< 0.001), L (OR = 1.4, 95% CI: 1.1–1.7, *p *< 0.001), PLT (OR = 1.0, 95% CI: 1.0–1.0, *p *= 0.02), D-dimer (OR = 1.0, 95% CI: 1.0–1.0, *p *< 0.001), PT (OR = 0.7, 95% CI: 0.5–1.0, *p *= 0.02), and RDW (OR = 6.4, 95% CI: 2.9–13.9, *p *< 0.001) were significantly associated with CAL in acute stage KD.

**Table 2:** Univariate and multivariate logistic regression analysis of independent factors for CAL in acute stage KD.

Independent factors	Univariate analysis		Multivariate analysis	
	OR (95% CI)	*p* value	OR (95% CI)	*p* value
WBC (×10^9^/L)	1.3 (1.1–1.4)	<0.001	1.1 (0.9–1.3)	0.2
L (×10^9^/L)	1.4 (1.1–1.7)	<0.001	1.2 (0.9–1.7)	0.2
RDW (%)	6.4 (2.9–13.9)	<0.001	7.0 (2.6–19.2)	<0.001
PLT (×10^9^/L)	1.0 (1.0–1.0)	0.02	1.0 (0.998–1.010)	0.2
PT (s)	0.7 (0.5–1.0)	0.02	0.4 (0.2–0.8)	0.01
D-dimer (mg/L)	1.0 (1.0–1.0)	<0.001	1.0 (1.004–1.012)	<0.001

KD, Kawasaki disease; CAL, coronary artery lesion; WBC, white blood cell count; L, lymphocyte count; RDW, red blood cell distribution; PLT, platelet count; PT, prothrombin time; OR, o/*ds ratio; 95% CI, 95% confidence interval.

Multivariable logistic regression analysis showed that D-dimer (OR = 1.0, 95% CI: 1.004–1.012, *p *< 0.001), PT (OR = 0.4, 95% CI: 0.2–0.8, *p *= 0.01), and RDW (OR = 7.0, 95% CI: 2.6–19.2, *p *< 0.001) were independent predictors of CAL in acute stage KD.

### Correlation analysis

The CAL in acute stage KD was positively correlated with D-dimer (*r* = 0.4, *p *< 0.001) and RDW (*r* = 0.5, *p *< 0.001), but negatively correlated with PT (*r* = −0.2, *p *< 0.05).

### ROC analysis

**Figure 1:**
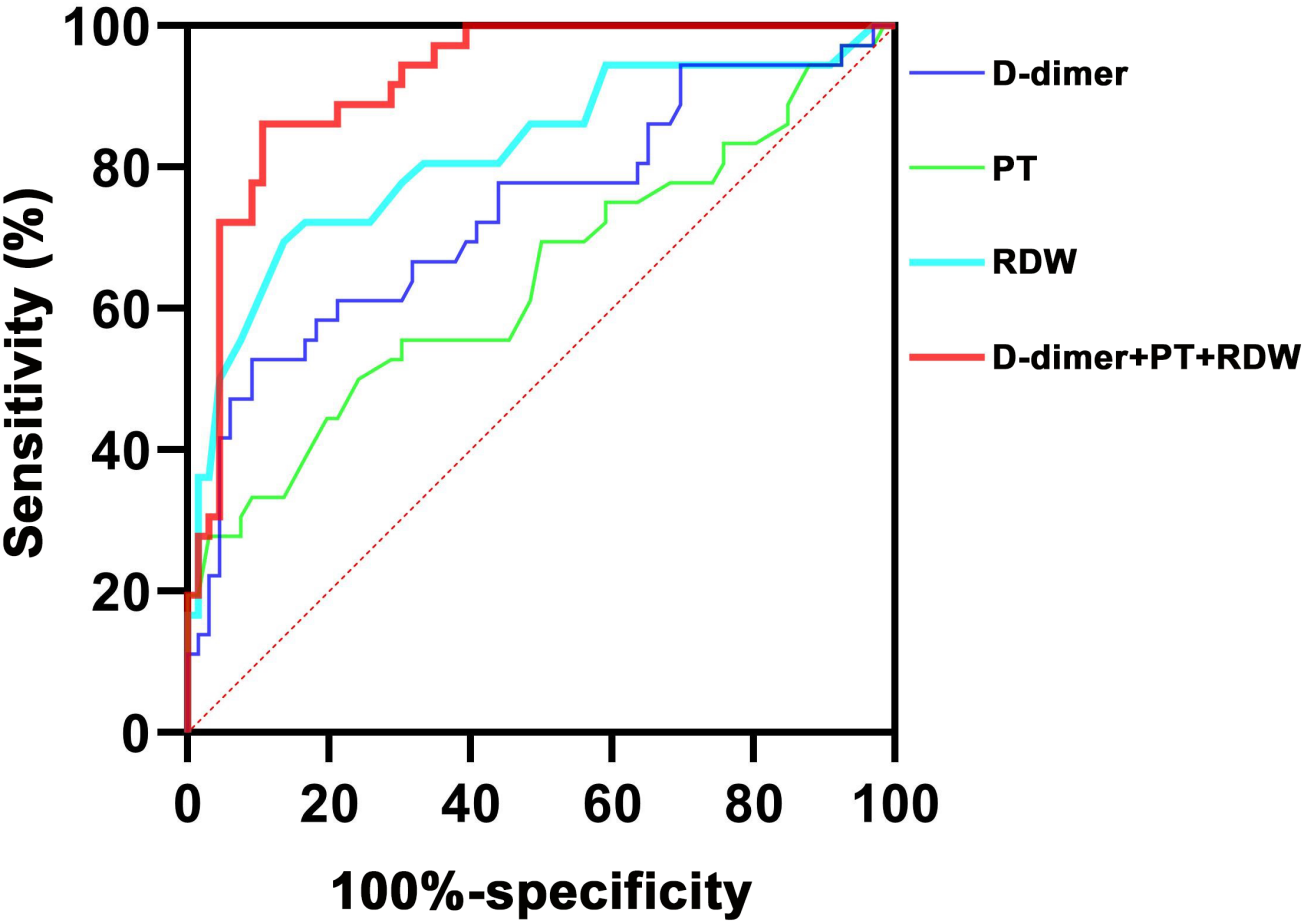
ROC curves of using different indicators to predict CAL in children with acute stage KD. ROC, receiver operating characteristic; KD, Kawasaki disease; CAL, coronary artery lesion; RDW, red blood cell distribution; PT, prothrombin time.

The ROC curves of using D-dimer, PT, and RDW to predict CAL in children with acute stage KD were analyzed (Figure 1). The ROC curve analysis illustrated the best cutoff value for D-dimer of >511 ng/L, with an AUC of 0.736, a sensitivity of 52.8%, and a specificity of 90.9%. The best cutoff value of PT for CAL in acute stage KD was <13.2 s (AUC = 0.640), and its sensitivity and specificity were 75.8% and 50.0%, respectively. RDW cutoff value was determined to be >13.1%, and the AUC value was 0.819 with sensitivity and a specificity of 69.4% and 86.4%, respectively. Moreover, the ROC curve analysis results showed that the combination of the three indicators was the most valuable predictor of CAL in acute stage KD with an AUC of 0.922 (sensitivity, 86.1%; specificity, 89.4%).

## Discussion

Our study retrospectively evaluated the performances of D-dimer, PT, and RDW for the diagnosis of CAL in acute stage KD. Compared with KD children without CAL, KD children with CAL had significantly higher D-dimer and RDW levels, but lower PT levels. The results of logistic regression analysis and ROC curve analysis showed that D-dimer, PT, and RDW were independent predictors of CAL in children with acute stage KD. Spearman correlation analysis revealed that CAL positively correlated with D-dimer and RDW, and negatively correlated with PT. Besides, the combination of the three indicators had the highest diagnostic performance for CAL in acute stage KD with an AUC of 0.922, a sensitivity of 86.1%, and a specificity of 89.4%. These results suggested that the combination of D-dimer, PT, and RDW may serve in the identification of CAL in children with acute stage KD.

Previous studies have shown that excessive inflammation in the acute phase leading to vascular endothelial cell dysfunction is an important mechanism for the development of CAL (6, 25). RDW is a parameter representing the variations in the dimension of circulating erythrocytes (26). Several studies have documented that higher RDW levels are an independent predictor of mortality from various cardiovascular diseases (21, 22, 27). Our study showed that RDW was a predictor of CAL in children with acute stage KD. The cutoff value of RDW in our study was >13.1%, which is similar to the result reported in a retrospective study performed on 1,355 patients with KD, with a cutoff value of >14.55% (28). Nevertheless, the relationship between RDW and CAL in children with KD is unclear. The commonly accepted view is that factors during the development of CAL such as oxidative stress and inflammatory response may affect the production and maturation of erythrocytes, which in turn cause changes in the level of RDW (28, 29). D-dimer is a byproduct of fibrin degradation and is widely recognized as a biomarker of thromboembolism (30). Compared with the NCAL group, our children in the CAL group had significantly higher D-dimer levels. Consistently, Zhou et al. also found a significant increase in serum D-dimer levels in KD children complicated with CAL (31). PT is a measure of coagulation status, and low PT levels have been identified as a risk factor related to major adverse cardiovascular events (18, 32). Our study showed that KD children with CAL had significantly lower PT levels than those without CAL, which was in line with those reported by Huang et al. (33). Importantly, the D-dimer and PT levels were firstly identified as predictors of CAL in acute stage KD in the present study. Overall, the present study demonstrated the diagnostic performances of D-dimer, PT, and RDW for CAL in children with acute stage KD.

Notably, this study is the first to evaluate the combined diagnostic performance of D-Dimer, PT, and RDW for CAL in children with acute stage KD. Our results showed that the combination of the three indicators had the highest diagnostic performance for CAL in acute stage KD, compared with D-dimer, PT, and RDW alone. These results suggested that RDW and D-dimer elevation combined with PT reduction could be used clinically as important indicators to predict the risk of CAL, thus providing some clinical reference for early treatment and prevention of CAL in acute stage KD. According to our findings, the WBC and PLT levels in KD children with CAL were also significantly higher than that in children without CAL. However, the diagnostic performances of WBC and PLT for CAL in children with acute stage KD were not established in our study. In contrast, Liu et al. found that WBC > 20 × 10^9^/L and PLT > 450 × 10^9^/L were risk factors for KD complicated with CAL by analyzing the clinical data of 485 KD children in Hangzhou (34). The reason for the discrepant results may be the bias introduced by the difference in sample size and region as well as non-standardization of laboratory testing methods. Thus, further studies with a large sample size are needed to validate these findings.

There were several limitations to our study. First, our study was performed at a single institution, adopting a retrospective design. Second, the relatively low sample size of KD children with CAL may lead to bias in the analysis. Third, all subjects were Chinese, leading to the possibility that our findings are not directly generalizable to other populations. Fourth, the severity or morphological characteristics of CALs were not assessed owing to the retrospective design of the study. Therefore, further multicenter studies with a prospective design and larger sample size should be conducted to confirm the Diagnostic performances of D-dimer, PT, and RDW for CAL in children with acute stage KD.

## Conclusion

In conclusion, children with CAL had significantly higher D-dimer and RDW levels, but lower PT levels than those without CAL. The D-dimer, RDW, and PT may help predict CAL in children with acute stage KD. A combination of D-dimer, PT, and RDW may be useful as a screening tool for CAL in acute stage KD. However, further prospective studies are needed to determine the optimal cutoff values for the three indicators.

## Data Availability

The raw data supporting the conclusions of this article will be made available by the authors, without undue reservation.

## References

[1] Menikou S Langford PR Levin M. Kawasaki disease: the role of immune complexes revisited. Front Immunol. (2019) 10:1156. 10.3389/fimmu.2019.01156 31263461 PMC6584825

[2] Rife E Gedalia A. Kawasaki disease: an update. Curr Rheumatol Rep. (2020) 22(10):75. 10.1007/s11926-020-00941-4 32924089 PMC7487199

[3] Huang X Huang P Zhang L Xie X Xia S Gong F Is aspirin necessary in the acute phase of kawasaki disease? J Paediatr Child Health. (2018) 54(6):661–4. 10.1111/jpc.13816 29271519

[4] Lu Y Guo Y Si F Chen T Jin M Wen Y Predictive value of heart rate deceleration capacity on coronary artery lesion in acute phase of kawasaki disease. Sci Rep. (2020) 10(1):10211. 10.1038/s41598-020-67121-3 32576944 PMC7311450

[5] Butters C Curtis N Burgner DP. Kawasaki disease fact check: myths, misconceptions and mysteries. J Paediatr Child Health. (2020) 56(9):1343–5. 10.1111/jpc.15101 32770807

[6] Zhang D Liu L Huang X Tian J. Insights into coronary artery lesions in kawasaki disease. Front Pediatr. (2020) 8:493. 10.3389/fped.2020.00493 32984207 PMC7477115

[7] Tsuda E Singhal M. Role of imaging studies in kawasaki disease. Int J Rheum Dis. (2018) 21(1):56–63. 10.1111/1756-185x.13210 29115035

[8] van Stijn D Planken N Kuipers I Kuijpers T. Ct angiography or cardiac mri for detection of coronary artery aneurysms in kawasaki disease. Front Pediatr. (2021) 9:630462. 10.3389/fped.2021.630462 33614558 PMC7889592

[9] Jun H Ko KO Lim JW Yoon JM Lee GM Cheon EJ. Age-Adjusted plasma N-terminal pro-brain natriuretic peptide level in kawasaki disease. Korean J Pediatr. (2016) 59(7):298–302. 10.3345/kjp.2016.59.7.298 27588030 PMC5007425

[10] Kaneko K Yoshimura K Ohashi A Kimata T Shimo T Tsuji S. Prediction of the risk of coronary arterial lesions in kawasaki disease by brain natriuretic peptide. Pediatr Cardiol. (2011) 32(8):1106–9. 10.1007/s00246-011-9986-8 21487793

[11] Kim MK Song MS Kim GB. Factors predicting resistance to intravenous immunoglobulin treatment and coronary artery lesion in patients with kawasaki disease: analysis of the Korean nationwide multicenter survey from 2012 to 2014. Korean Circ J. (2018) 48(1):71–9. 10.4070/kcj.2017.0136 29171205 PMC5764872

[12] Yu HR Kuo HC Huang EY Liang CD Hwang KP Lin IC Plasma clusterin levels in predicting the occurrence of coronary artery lesions in patients with kawasaki disease. Pediatr Cardiol. (2010) 31(8):1151–6. 10.1007/s00246-010-9769-7 20711835

[13] Zheng X Zhang Y Liu L Yue P Wang C Zhou K N-Terminal pro-brain natriuretic peptide as a biomarker for predicting coronary artery lesion of kawasaki disease. Sci Rep. (2020) 10(1):5130. 10.1038/s41598-020-62043-6 32198398 PMC7083930

[14] Wan J Yang X He W Zhu Y Zhu Y Zeng H Serum D-dimer levels at admission for prediction of outcomes in acute pancreatitis. BMC Gastroenterol. (2019) 19(1):67. 10.1186/s12876-019-0989-x 31046705 PMC6498652

[15] Rad F Dabbagh A Dorgalaleh A Biswas A. The relationship between inflammatory cytokines and coagulopathy in patients with COVID-19. J Clin Med. (2021) 10(9):2020. 10.3390/jcm10092020 34065057 PMC8125898

[16] Demirkol S Balta S Cakar M Unlu M Arslan Z Kucuk U. Red cell distribution width: a novel inflammatory marker in clinical practice. Cardiol J. (2013) 20(2):209. 10.5603/cj.2013.0037 23558882

[17] Arnold N Lechner K Waldeyer C Shapiro MD Koenig W. Inflammation and cardiovascular disease: the future. Eur Cardiol. (2021) 16:e20. 10.15420/ecr.2020.50 34093741 PMC8157394

[18] Peng J Liu MM Liu HH Guo YL Wu NQ Dong Q Association of circulating proprotein convertase subtilisin/kexin type 9 concentration, prothrombin time and cardiovascular outcomes: a prospective cohort study. Thromb J. (2021) 19(1):90. 10.1186/s12959-021-00344-0 34809656 PMC8607723

[19] Soomro AY Guerchicoff A Nichols DJ Suleman J Dangas GD. The current role and future prospects of D-dimer biomarker. Eur Heart J Cardiovasc Pharmacother. (2016) 2(3):175–84. 10.1093/ehjcvp/pvv039 27533759

[20] Lan W Liu E Sun D Li W Zhu J Zhou J Red cell distribution in critically ill patients with chronic obstructive pulmonary disease. Pulmonology. (2022):S2531-0437(22)00082-4. 10.1016/j.pulmoe.2022.04.001 35501276

[21] Zorlu A Bektasoglu G Guven FM Dogan OT Gucuk E Ege MR Usefulness of admission red cell distribution width as a predictor of early mortality in patients with acute pulmonary embolism. Am J Cardiol. (2012) 109(1):128–34. 10.1016/j.amjcard.2011.08.015 21958741

[22] Aung N Ling HZ Cheng AS Aggarwal S Flint J Mendonca M Expansion of the red cell distribution width and evolving iron deficiency as predictors of poor outcome in chronic heart failure. Int J Cardiol. (2013) 168(3):1997–2002. 10.1016/j.ijcard.2012.12.091 23351789

[23] McCrindle BW Rowley AH Newburger JW Burns JC Bolger AF Gewitz M Diagnosis, treatment, and long-term management of kawasaki disease: a scientific statement for health professionals from the American heart association. Circulation. (2017) 135(17):e927–e99. 10.1161/cir.0000000000000484 28356445

[24] The Subspecialty of Cardiology tSoP, Chinese Medical Association, The Editorial Board CJoP. Recommendations for clinical management of coronary artery disease in kawasaki disease (2020 revision). Chin J Pediatr. (2020) 58(09):718–24. 10.3760/cma.j.cn112140-20200422-00421 32872711

[25] Agarwal S Agrawal DK. Kawasaki disease: etiopathogenesis and novel treatment strategies. Expert Rev Clin Immunol. (2017) 13(3):247–58. 10.1080/1744666x.2017.1232165 27590181 PMC5542821

[26] Koma Y Onishi A Matsuoka H Oda N Yokota N Matsumoto Y Increased red blood cell distribution width associates with cancer stage and prognosis in patients with lung cancer. PloS one. (2013) 8(11):e80240. 10.1371/journal.pone.0080240 24244659 PMC3823700

[27] Veeranna V Zalawadiya SK Panaich S Patel KV Afonso L. Comparative analysis of red cell distribution width and high sensitivity C-reactive protein for coronary heart disease mortality prediction in multi-ethnic population: findings from the 1999–2004 Nhanes. Int J Cardiol. (2013) 168(6):5156–61. 10.1016/j.ijcard.2013.07.109 24016543

[28] Ming L Cao HL Li Q Yu G. Red blood cell distribution width as a predictive marker for coronary artery lesions in patients with kawasaki disease. Pediatr Cardiol. (2021) 42(7):1496–503. 10.1007/s00246-021-02633-x 34036412 PMC8463334

[29] Semba RD Patel KV Ferrucci L Sun K Roy CN Guralnik JM Serum antioxidants and inflammation predict red cell distribution width in older women: the women’s health and aging study I. Clin Nutr. (2010) 29(5):600–4. 10.1016/j.clnu.2010.03.001 20334961 PMC3243048

[30] Mishra Y Pathak BK Mohakuda SS Tilak T Sen S P H Relation of D-dimer levels of COVID-19 patients with diabetes mellitus. Diabetes Metab Syndr. (2020) 14(6):1927–30. 10.1016/j.dsx.2020.09.035 33035824 PMC7528836

[31] Zhou Y Wang S Zhao J Fang P. Correlations of complication with coronary arterial lesion with vegf, plt, D-dimer and inflammatory factor in child patients with kawasaki disease. Eur Rev Med Pharmacol Sci. (2018) 22(16):5121–6. 10.26355/eurrev_201808_15706 30178831

[32] Solms A Frede M Berkowitz SD Hermanowski-Vosatka A Kubitza D Mueck W Enhancing the quality of rivaroxaban exposure estimates using prothrombin time in the absence of pharmacokinetic sampling. CPT. (2019) 8(11):805–14. 10.1002/psp4.12444 PMC6875705 31276324

[33] Huang J-M Wu Y-Z Lai W-Q Sheng X-M Yu L-Z. Changes and clinical observation in plasma brain natriuretic peptide, D-dimer and fibrinogen levels in children with kawasaki disease during the acute phase. J Pract Med. (2015) 31(12):3. 10.3969/j.issn.1006-5725.2015.12.034

[34] Liu F Wu K-Y. Epidemiological survey and analysis of risk factors for concurrent coronary artery damage in children with kawasaki disease in Hangzhou from 2014−2018. Public Health Management China. (2020) 36(5):5. 10.19568/j.cnki.23-1318.2020.05.009

